# Sanatoria for Consumption

**Published:** 1901-04-20

**Authors:** 


### SANATORIA FOR CONSUMPTION.

Altadore Sanatorium, Kilpedder, co. Wicklow.—Among Wicklow Mountains, 750 feet above sea-level; faces south, sheltered from north, east, and west. Accommodation for 9 patients, with outdoor shelters. Medical officer: Dr. J. C. Smyth. Special feature: Nordrach treatment. Nearest railway station, Greystones, Wicklow and Wexford Railway; carriage to meet patients.

Approximate fees, 4 guineas per week.

"Belle Vue" Sanatorium, Shotley Bridge, co. Durham.—Upon a slope of the Derwent Valley, 500 feet above sea-level. Accommodation for 10 patients; conservatory 30 feet by 20 feet (hot water pipes); outdoor shelters and sleeping cabins. Medical officer: Dr. E. W. Diver. Special feature: Open-air treatment. Nearest railway station, Shotley Bridge (one mile).

Approximate fees, from 3 to 5 guineas weekly.

Brinklea Sanatorium, Bournemouth, W.—Verging on Branksome Park. Accommodation for 12 patients. Medical officers: Dr. Kinsey-Morgan, Dr. Kinsey-Morgan, jun. Special feature: Open-air treatment. Nearest railway station, Bournemouth West.

Approximate fees per week, 4 to 6 guneas.

Brookside, Clare, Suffolk.—Accommodation for 12 patients. Medical director: Dr. Jane Walker; Resident medical officer: Miss Sharp, M.B. Special feature: Openair treatment. Communications to Dr. Jane Walker, 62 Gower Street, W.C., or Mrs. Wilson, Brookside, Clare, Suffolk. Nearest railway station, Clare.

Approximate fees, 2 guineas per week.

The Consumptive Sanatoria of Scotland, Bridge of Weir, Renfrewshire, N.B.—260 feet above sea-level; aspect to the south. Present accommodation for 40 females; will shortly be doubled. Medical officer: Dr. Thompson Campbell. Special features of treatment: Nordrach system. Nearest railway station, Bridge of Weir.

Free to female patients above 16 years, who are inhabitants of Scotland.

Cotswold Sanatorium, near Stroud, Gloucestershire.—On the Cotswold Hills; elevation, 800 feet. Medical officers: Drs. S. T. Pruen and C. Braine-Hartnell. Treatment: On the Nordrach lines. Communications to the Secretary, 2 Ormond Place, Cheltenham. Railway stations, Cheltenham, Gloucester, and Stroud.

Approximate fees, 4 and 5 guineas per week.

Crooksbury Sanatorium, Farnham, Surrey.—On the south slope of Crooksbury Ridges. Accommodation for 12, being enlarged to 20. Medical officers: Drs. F. R. Walters and C. G. Higginson. Special feature: Thorough Nordrach treatment. Railway stations, most convenient, Farnham, S.W.R., and Ash, S.E.R.

Approximate fees per week, from $4\frac{1}{2}$ guineas. Reduction in suitable cases for prolonged stay.

Dunstone Park Home, Marldon Hill, Paignton, South Devon.—550 feet above sea-level; aspect south and south-west. Accommodation for eight patients. Visiting physician, Dr. James Alexander. Patients can be attended by doctors of their own choice. Treatment: Open-air, rest, liberal diet.

Nearest railway station: Paignton, $\frac{1}{2}$ mile.

East Anglian Sanatorium, Broomfields, Nayland, Suffolk.—Accommodation for 35 patients. Medical director: Dr. Jane Walker. Resident medical officer: Dr. Mabel Paine. Special features: Treatment by open-air, feeding, carefully arranged rest and exercise. Communications to Dr. Jane Walker, 62 Gower Street, W.C. Nearest railway station, Bures, G.E.R.

Approximate fees per week, 4 to 6 guineas.

The Firs, Mundesley, Norfolk. Within $\frac{1}{4}$ mile of the sea. Accommodation for 7 patients. Medical officers: Dr. Burton Fanning and Mr. W. J. Fanning. Open-air treatment. Nearest railway station, Mundesley, G.E.R. and Mid. and G.N.

Approximate fees per week, 3 guineas.

The Forster Green Hospital, Fortbreda, near Belfast.—Situate in the Castlereagh Hills. Beds 30. Medical officers: Drs. Purdon, Simpson, and Howard Sinclair. Treatment: On open-air principles. Communications to the Secretary, 2 May Street, Belfast.

Approximate fees per week. Intended for the poor, some beds being quite free.

Hailey Open-air Sanatorium, Ipsden, near Wallingford, on the Chiltern Hills.—The altitude of the grounds ranging from 300 to 600 feet above sea-level. Accommodation: 20 beds, 8 isolated sleeping châlets, commodious dining and reception rooms. Medical officers: Drs. Charles Reinhardt and Ronald Macfie. Treatment: Open-air and super-nutrition, climatic. Nearest railway station, Goring, G.W.R.

Approximate fees, £4 4s. to £6 6s. per week.

Inglewood Private Sanatorium, St. Lawrence, Isle of Wight.—Accommodation for 17 patients. Special features: Open-air treatment; night shelters, facing south. Medical officer: Dr. J. A. Chowry-Muthu. Nearest railway station, Ventnor, *viâ* Portsmouth and Ryde.

Approximate fees per week, $3\frac{1}{2}$ to 4 guineas.

Knocksualtach Sanatorium, Kirkmichael, Blairgowrie, N.B.—In the Highlands of Perthshire. Accommodation for 6 ladies. Medical officer: Dr. Mary F. Nannetti. Treatment: Strictly on open-air and liberal diet principles. Nearest railway station, Blairgowrie.

Approximate fees, £4 4s. to £5 5s. per week.

Maitland House, Kidmore, Reading.—Situated in the district of Chiltern Hills, 400 feet above sea level, on gravel soil. Accommodation for 10 patients; outdoor shelters. Medical officer: Dr. Esther Colebrook; Consultant: Dr. H. Gilford. Treatment: Regulated rest and exercise, in continual fresh air. Nearest railway station, Reading.

Approximate fees 3 guineas per week.

Mendip Hills Sanatorium, Hill Grove, over Wells Somerset. 850 feet above sea-level in thickly-wooded grounds of 160 acres; though standing so high, the house is surrounded by still higher ground, and so sheltered from gales; large fir plantations intersected by walks. Accommodation for 18 patients. Medical officer: Dr. Charles J. Whitby. Treatment: Open-air. Railway stations, Wells (Great Western Railway), Binegar (Somerset and Dorset).

Approximate fees, 5 guineas per week.

Moorcote Sanatorium, Eversley, Hants.—Situated in a district richly wooded with pines. Accommodation for 12 patients with outdoor shelters. Medical officer: Dr. C. Maberley. Treatment: Nordrach. Nearest railway station, Wellington College.

The Mundesley Sanatorium, Cook's Hill, Mundesley, Norfolk.—One mile from Mundesley. Accommodation for 18 patients. Visiting physician: Dr. F. W. Burton Fanning; resident physician: Dr. W. J. Fanning. Treatment: Open-air. Nearest railway station, Mundesley.

Approximate fees per week, £5 5s.

Nordrach-on-Dee, Banchory, N.B.—Near Banchory, Middle Deeside. In the middle of a pine forest. Accommodation for 42 patients. Medical officers: Drs. David Lawson, senior physician; Noel D. Bardswell, John E. Chapman, laboratist. Treatment on modified Nordrach lines (research laboratory). Nearest railway station, Banchory, N.B.

Approximate fees, 5 guineas weekly.

Nordrach-upon-Mendip, Blagdon, Bristol.—862 feet above sea; on Mendip Hills, Somerset. 40 bed and reception rooms. Medical officers: Drs. Rowland Thurnam and Neville C. Gwynn. Treatment: Strictly Nordrach. Railway stations, Yatton or Cheddar, Great Western Railway.

Approximate fees per week, 5 guineas.

Overton Hall, Poole Road, Bournemouth.—Accommodation for 16 patients, with out-door shelters. Medical officers: Drs. F. Pott and C. Guthrie Stein. Treatment: Open-air and super-nutrition. Communications: Bournemouth West.

Approximate fees per week, 5 guineas; medical fee, 1 guinea.

Rostrevor Sanatorium, co. Down, Ireland.—Situated on the Mourne Mountains, 350 feet above sea. Accommodation for 18 patients. Medical officer: Dr. F. Howard Sinclair. Treatment: Combination of Nordrach and Falkenstein. Nearest railway station, Warrenpoint, Great Northern Railway, Ireland.

Approximate fees per week, 3½ guineas.

Rudgwick Sanatorium, Sussex.—Situated on the south side of the Surrey hills, 400 feet above sea-level. Accommodation for 13 patients. Visiting medical officer: Dr. Annie McCall. Treatment: Open-air. The treatment of children is a special feature at this sanatorium. Nearest railway station, Rudgwick.

Approximate fees (adults and children) per week, 2½ guineas.

"The Sanatorium," St. Michael's, near Ashford, Kent.—200 feet above sea-level, surrounded by extensive woods of pine, fir, and other trees. Accommodation for 17 patients. Visiting medical officer: Dr. Peter Paget. Treatment: Open-air. Railway stations, Tenterden and Headcorn.

Approximate fees per week, 3½ guineas.

Stourfield Park Sanatorium, Pokesdown, Bournemouth.—Accommodation for 40 patients, with winter garden, 10 acres of grounds, and numerous specially constructed chalets. Medical officer: Dr. D. Thompson. Treatment: Modified Nordrach. Communications to the Sanatorium or Percival Turner, 4 Adam Street, Adelphi, London, W.C. Railway stations, Boscombe and Bournemouth.

Approximate fees, from 4 guineas per week.

Sunny Nook, Grange-over-Sands. — Southern aspect, sheltered from north and east winds by cliff. Accommodation for 4 patients, with outdoor shelters. Treatment: Dietary and nursing. Nearest railway station, Grange-over-Sands.

Approximate fee from 42s. per week.

Swiss Villa Sanatorium, Swanage, Dorset.—On sea cliffs. Accommodation for 4 patients; garden shelter. Medical officer: Dr. J. M. Barbour. Treatment: Nordrach. Nearest railway station, Swanage.

Approximate fees per week, four to six guineas.

Victoria Hospital for Consumption, Craigleith, Edinburgh.—Situation, one mile north-west of Edinburgh, on a slope facing due south. For the gratuitous treatment of consumptives strictly on the open-air principle. Accommodation for 23 patients. Senior physician: Dr. R. W. Philip.

Whitmead Hill Sanatorium, Farnham.—Stands in 19 acres of pines and heather in the Hindhead district of Surrey, sheltered from north by Crooksbury Hill. Accommodation for 18 patients. Medical officer: Dr. J. Hurd Wood. Treatment: Open-air. Railway station, Farnham, South-Western Railway.

Approximate fees, £5 5s. per week.

Woodburn Sanatorium, Morningside, Edinburgh.—At the foot of the Braid Hills, southern exposure on a sandy slope. Accommodation for 17 patients; winter garden; natural shelters for resting outdoors. Medical officers: Drs. Jas. J. Galbraith and W. P. Mears; consulting medical officer: Dr. R. W. Philip. Treatment: Strict open-air, liberal diet. Nearest railway station, Edinburgh Suburban Morningside Station.

Approximate fees, 5 guineas per week inclusive.

### HYDROPATHIC AND OTHER ESTABLISHMENTS.

Alexandra Hospital, Woodhall Spa, Lincolnshire.—Accommodation: 28 beds. Medical officers: Drs. C. J. Williams and H. W. Gwyn. Treatment: By baths and mineral waters. Nearest railway station, Woodhall Spa.

Approximate fees per week, 10s.

